# EGFR-directed antibodies increase the risk of severe infection in cancer patients

**DOI:** 10.1186/s12916-015-0276-9

**Published:** 2015-02-20

**Authors:** Mehmet Altan, Barbara Burtness

**Affiliations:** Yale University School of Medicine and Yale Cancer Center, 333 Cedar Street, WWW225, New Haven, CT 06520-8028 USA

**Keywords:** EGFR, Infection, Monoclonal antibody

## Abstract

Monoclonal antibodies directed to the epidermal growth factor receptor (EGFR) have a role in the management of several solid tumors, alone or in combination with chemotherapy or radiation therapy. Recognized toxicities have included hypersensitivity reactions, rash, hypomagnesemia, and constitutional symptoms, but the possibility that the agents lead to immunosuppression or increase the risk of infection has only recently been recognized. Two latest meta-analyses, including the recently published article by Qi et al., highlight the increased risk of severe infections with EGFR-directed monoclonal antibodies. Further studies are needed to better identify the association between EGFR-directed monoclonal antibody treatment and infection, as well as to elucidate the mechanism of this toxicity and to develop tools to identify patients at increased risk for these complications. In the meantime, awareness of the role of EGFR-directed antibodies in increased infection risk may have implications for dose modification strategies in both clinical trial design and the practice of oncology.

Please see related article: http://www.biomedcentral.com/1741-7015/12/203.

## Background

Monoclonal antibodies targeted to human epidermal growth factor receptor (HER) family members, such as the epidermal growth factor receptor (EGFR) and HER-2, are widely used in the management of patients with solid tumors. Trastuzumab and pertuzumab have significantly improved outcomes for patients with HER-2 amplified breast cancer [1], while the EGFR-directed antibody cetuximab improves response and survival in patients with head and neck cancer [2-4] and cetuximab and the EGFR-directed antibody panitumumab prolong survival in selected patients with colorectal cancer [5,6]. Recognized toxicities of trastuzumab have included cardiac dysfunction, diarrhea, and infusion reactions, whilst hypomagnesemia [2], rash [7], and hypersensitivity reactions [8] have been reported for cetuximab. Until recently, the infections observed during treatment with EGFR- or HER-2-directed antibodies had not been attributed to the administration of the antibodies themselves. However, meta-analyses and path-breaking preclinical studies indicating a potential role for EGFR in regulation of innate immunity, now call for a reexamination of the evidence and greater vigilance in future trials of these agents.

## Risk of infection after EGFR-directed antibody therapy

Qi et al. [9] have recently published a meta-analysis in *BMC Medicine* on the incidence and risk of severe infections in cancer patients treated with EGFR-directed antibody therapy. Their analysis of 14,066 patients in 26 randomized controlled trials demonstrates an increased risk for severe infection, with a hazard ratio (HR) of 1.34 (95% CI: 1.10–1.62, *P* = 0.003), and a numerical but not statistically significant increase in fatal infections [9]. This effect was most readily determined in the cancers for which EGFR-directed antibody therapy is most common, colorectal cancer, head and neck cancer, and non-small cell lung cancer, perhaps reflecting greater power in these analyses. The early recognition of an increased incidence of neutropenia in E5397, a randomized trial of cisplatin/placebo or cisplatin/cetuximab, was seemingly explained by the greater exposure to cytotoxic chemotherapy in patients on the cetuximab arm [2]. In that trial, neutropenia increased from 14% to 30% with the addition of cetuximab (*P* = 0.04), but the number of treatment cycles was associated with risk of hematologic toxicity and the difference between the arms was not significant when duration of chemotherapy exposure was controlled for. The current study undertook a meta-regression analysis to address the possibility that increased infection resulted when better anticancer efficacy prolonged the duration of exposure to both the EGFR inhibitor and chemotherapy, and found that longer duration of therapy actually predicted for a significantly lower risk of severe infection.

The findings of Qi et al. [9] align well with two other recent meta-analyses which also demonstrate an increase in the risk of infection after HER family-directed antibodies. Funakoshi et al. [10] also undertook a meta-analysis of trials with cetuximab or panitumumab in solid tumor patients. Their analysis included 14,957 patients in 28 randomized controlled trials; interestingly, the two meta-analyses include an overlapping but not identical set of trials, and thus may be seen as confirmatory of each other. There are 17 trials included in both analyses, with an additional 9 included only in the Qi paper [9], and an additional 11 only in the Funakoshi paper [10]. The current paper includes several trials in which dual targeted therapy is tested, e.g., bevacizumab plus cetuximab or bortezomib plus cetuximab, which may introduce as yet undefined effects from other targeted therapies; however, the negative sensitivity analysis is reassuring that the effect is not largely a reflection of targeted agents other than EGFR-directed antibodies. Confirmatory data also come from trials with unapproved agents, as these were excluded from both the Qi and Funakoshi analyses, but similar effects have been described, including a 14% rate of infection after therapy with the humanized anti-EGFR-antibody zalutumumab [11,12]. Additionally, Funakoshi et al. [13] have described an increased risk of high grade infection (HR, 1.21) and febrile neutropenia (HR, 1.28) in a meta-analysis of 10,094 patients in 13 randomized controlled trials of the HER2-directed antibodies trastuzumab and/or pertuzumab [13].

The mechanism of action for this effect has not been established; however, recent studies demonstrating a role for EGFR in regulation of innate immunity suggest that down-regulation of EGFR-dependent signaling in normal tissues may explain an increase in severe infection. Host cells are equipped with cellular sensors which detect specific microbial components and activate cellular antimicrobial response. Toll-like receptors (TLRs) are an important class of such sensors, expressed in macrophages and dendritic cells. Activation of TLR-dependent signaling results in the synthesis of protective antimicrobial molecules such as interferon [14]. TLR-3 requires tyrosine phosphorylation to recruit adaptor proteins, and this process has recently been described to depend on EGFR activation and Src binding [14]; inhibition of EGFR was demonstrated to permit increased viral replication. Dysregulated EGFR function in normal respiratory epithelium and dendritic cells could thus be implicated in the increased risk of severe infection following cetuximab, panitumumab, or zalutumumab therapy, and may also explain excess fulminant infections when EGFR inhibition is added to mTOR inhibition in attempted synthetic lethal cancer therapy, as described in several clinical trials [15].

## Conclusions

Further preclinical studies to determine the role EGFR signaling plays in innate immunity will be of interest. In the meantime, awareness that EGFR-directed antibodies increase infection risk mandates greater caution when administering these agents in patients with active infection, and has implications for the monitoring, dose modification schemes, and correlative trials which are appropriate to future trials with these agents. Moreover, synthetic lethal drug combinations will need to be evaluated carefully for the possibility of additive or synergistic effects in suppression of innate immunity when EGFR inhibitors are in the mix.

## Abbreviations

EGFR: Epidermal growth factor receptor
HER: Human epidermal growth factor receptor
HR: Hazard ratio
TLRs: Toll-like receptors

## References

[1] Arteaga CL, Engelman JA (2014). ERBB receptors: from oncogene discovery to basic science to mechanism-based cancer therapeutics. Cancer Cell..

[2] Burtness B, Goldwasser MA, Flood W, Mattar B, Forastiere AA (2005). Phase III randomized trial of cisplatin plus placebo compared with cisplatin plus cetuximab in metastatic/recurrent head and neck cancer: an Eastern Cooperative Oncology Group study. J Clin Oncol..

[3] Bonner JA, Harari PM, Giralt J, Azarnia N, Shin DM, Cohen RB (2006). Radiotherapy plus cetuximab for squamous-cell carcinoma of the head and neck. N Engl J Med..

[4] Vermorken JB, Mesia R, Rivera F, Remenar E, Kawecki A, Rottey S (2008). Platinum-based chemotherapy plus cetuximab in head and neck cancer. N Engl J Med..

[5] Bokemeyer C, Van Cutsem E, Rougier P, Ciardiello F, Heeger S, Schlichting M (2012). Addition of cetuximab to chemotherapy as first-line treatment for KRAS wild-type metastatic colorectal cancer: pooled analysis of the CRYSTAL and OPUS randomised clinical trials. Eur J Cancer..

[6] Price TJ, Peeters M, Kim TW, Li J, Cascinu S, Ruff P (2014). Panitumumab versus cetuximab in patients with chemotherapy-refractory wild-type KRAS exon 2 metastatic colorectal cancer (ASPECCT): a randomised, multicentre, open-label, non-inferiority phase 3 study. Lancet Oncol..

[7] Saltz LB, Meropol NJ, Loehrer PJ, Needle MN, Kopit J, Mayer RJ (2004). Phase II trial of cetuximab in patients with refractory colorectal cancer that expresses the epidermal growth factor receptor. J Clin Oncol..

[8] Chung CH, Mirakhur B, Chan E, Le QT, Berlin J, Morse M (2008). Cetuximab-induced anaphylaxis and IgE specific for galactose-alpha-1,3-galactose. N Engl J Med..

[9] Qi WX, Fu S, Zhang Q, Guo XM (2014). Incidence and risk of severe infections associated with anti-epidermal growth factor receptor monoclonal antibodies in cancer patients: a systematic review and meta-analysis. BMC Med..

[10] Funakoshi T, Suzuki M, Tamura K (2014). Infectious complications in cancer patients treated with anti-EGFR monoclonal antibodies cetuximab and panitumumab: a systematic review and meta-analysis. Canc Treat Rev.

[11] Machiels JP, Subramanian S, Ruzsa A, Repassy G, Lifirenko I, Flygare A (2011). Zalutumumab plus best supportive care versus best supportive care alone in patients with recurrent or metastatic squamous-cell carcinoma of the head and neck after failure of platinum-based chemotherapy: an open-label, randomised phase 3 trial. Lancet Oncol..

[12] Saloura V, Cohen EE, Licitra L, Billan S, Dinis J, Lisby S (2014). An open-label single-arm, phase II trial of zalutumumab, a human monoclonal anti-EGFR antibody, in patients with platinum-refractory squamous cell carcinoma of the head and neck. Cancer Chemother Pharmacol..

[13] Funakoshi T, Suzuki M, Muss HB (2015). Infection risk in breast cancer patients treated with trastuzumab: a systematic review and meta-analysis. Breast Canc Res Treat.

[14] Chattopadhyay S, Sen GC (2014). Tyrosine phosphorylation in Toll-like receptor signaling. Cytokine Growth Factor Rev.

[15] Burtness B, Marur S, Bauman JE, Golemis EA, Mehra R, Cohen SJ. Comment on “Epidermal growth factor receptor is essential for toll-like receptor 3 signaling”. Science Signaling. 2012;5(254):lc5. 10.1126/scisignal.2003734 23233526

